# Effect of dynamic compressive loading and its combination with a growth factor on the chondrocytic phenotype of 3-dimensional scaffold-embedded chondrocytes

**DOI:** 10.3109/17453670903413111

**Published:** 2009-12-04

**Authors:** Kosei Ando, Shinji Imai, Eiji Isoya, Mitsuhiko Kubo, Tomohiro Mimura, Suguru Shioji, Hisao Ueyama, Yoshitaka Matsusue

**Affiliations:** ^1^Department of Orthopaedic Surgery; ^2^Department of Biochemistry and Molecular Biology, Shiga University of Medical Science, Otsu, Shiga, Japan

## Abstract

**Background and purpose** Three-dimensionally (3D-) embedded chondrocytes have been suggested to maintain the chondrocytic phenotype. Furthermore, mechanical stress and growth factors have been found to be capable of enhancing cell proliferation and ECM synthesis. We investigated the effect of mechanical loading and growth factors on reactivation of the 3D-embedded chondrocytes.

**Methods** Freshly isolated chondrocytes from rat articular cartilage were grown in monolayer cultures and then in collagen gel. Real-time RT-PCR and histological analysis for aggrecan and type II and type I collagen was performed to evaluate their chondrocytic activity. Then, the 3D-embedded chondrocytes were cultured under either mechanical loading alone or in combination with growth factor. The dynamic compression (5% compression, 0.33 Hz) was loaded for 4 durations: 0, 10, 60, and 120 min/day. The growth factor administered was either basic fibroblast growth factor (bFGF) or bone morphogenetic protein-2 (BMP-2).

**Results** Mechanical loading statistically significantly reactivated the aggrecan and type II collagen expression with loading of 60 min/day as compared to the other durations. The presence of BMP-2 and bFGF clearly enhanced the aggrecan and type II collagen expression of 3D-embedded chondrocytes. Unlike previous reports using monolayer chondrocytes, however, BMP-2 or bFGF did not augment the chondrocytic phenotype when applied together with mechanical loading.

**Interpretation** Dynamic compression effectively reactivated the dedifferentiated chondrocytes in 3D culture. However, the growth factors did not play any synergistic role when applied with dynamic compressive loading, suggesting that growth factors should be administered at different time points during regeneration of the transplantation-ready cartilage.

## Introduction

Articular cartilage is characterized by its limited capacity for self-repair. The currently practiced forms of medical intervention to promote repair of the injured cartilage, e.g., drilling (Pridie 1959), microfracture (Rodrigo et al. 1994), or osteochondral graft (Matsusue et al. 1993), may not always lead to adequate repair (Newman 1998). Autologous chondrocyte implantation (ACI) was first applied clinically by Brittberg et al. (1994), and received much attention for its potential as a novel treatment of damaged cartilage. In many of the ACI protocols tried after the Brittberg report, the autologous chondrocytes have been prepared in monolayer culture and transplanted into the cartilage defects of the human joints. Several human clinical trials have, however, indicated that the reparative tissue generated in the ACI consists of fibrocartilage with limited amounts of hyaline cartilage (Knutsen et al. 2004).

Numerous authors have attributed the fibrocartilaginous quality of reparative tissue in the ACI to the dedifferentiation of chondrocytes prepared in monolayer culture. The chondrocytes cultured as monolayers have been found not to synthesize the extracellular matrix (ECM) (Holtzer et al. 1960, Abbot and Holtzer 1966, Mayne et al. 1976, von der Mark et al. 1977, Benya et al. 1978). A variety of attempts have been made to regenerate the transplantation-ready cartilage without losing chondrocytic phenotype. Chondrocytes three-dimensionally embedded in collagen have been suggested to maintain the chondrocytic phenotype for a relatively long time (Kimura et al. 1984, Uchio et al. 2000, Chaipinyo et al. 2004). Transplantation of the 3D-embedded chondrocytes has been undertaken in the hope of repairing damaged cartilage with better tissue (Ochi et al. 2001). The clinical validity of this method will be assessed in a near future, but the data accumulating from in vitro studies do not always favor the transplantation of 3D-cultured chondrocytes (Darling and Athanasiou 2005).

Other workers have used growth factors, which have been found to be capable of enhancing cell proliferation and ECM synthesis in vitro and in vivo. In most studies on the regeneration of transplantation-ready cartilage, recombinant growth factors have been tested individually or in combination. For example, basic fibroblast growth factor (bFGF) (Martin et al. 1999), bone morphogenetic protein-2 (BMP-2) (Sailor et al. 1996), insulin-like growth factor-I (IGF-I) (Guerne et al. 1994), and transforming growth factor-β1 (TGF−β1) (Malemud et al. 1991) have been used to enhance proliferation and differentiation in primary and subcultured chondrocytes.

Mechanical stress is another important factor that regulates the numerous aspects of chondrocytic activities (Broom et al. 1980, Palmoski and Brandt 1984, Schneiderman et al. 1986, Gray et al. 1988, Sah et al. 1989, Korver et al. 1992, Parkkinen et al. 1992, Guilak et al. 1994, Buschmann, et al. 1995, Lee and Bader 1997, Ragan et al. 1999, Elder et al. 2001). In vitro studies have shown that mechanical stimulation influences the ECM synthesis of cartilage explants (Broom et al. 1980, Palmoski et al. 1984, Schneiderman et al. 1986, Gray et al. 1988, Sah et al. 1989, Korver et al. 1992, Parkkinen et al. 1992, Guilak et al. 1994, Ragan et al. 1999) and of cultured chondrocytes (Buschmann et al. 1995, Lee and Bader 1997, Elder et al. 2001). As for the nature of mechanical loading, static compression has been shown to reduce ECM synthesis (Palmoski and Brandt 1984, Schneiderman et al. 1986, Gray et al. 1988, Sah et al. 1989, Ragan et al. 1999), whereas dynamic compression at low amplitude (1–5% compression loading, 0.01–1 Hz) stimulates the synthesis (Palmoski and Brandt 1984, Sah et al. 1989, Korver et al. 1992, Parkkinen et al. 1992, Buschmann, et al. 1995, Lee and Bader 1997, Elder et al. 2001).

We hypothesized that application of mechanical stress to the 3D-embedded chondrocytes would have a favorable effect in maintaining cartilaginous phenotype. Since a large number of chondrocytes must be prepared in monolayer culture in order to regenerate the transplantation-ready cartilage, the dedifferentiated chondrocytes must be reactivated. In the present study, we prepared 3D-embedded chondrocytes using cells cultured in monolayers. We investigated the chondrocytic phenotype at different stages of preparation. In order to reactivate the dedifferentiated chondrocytes, we applied dynamic mechanical loading to the 3D-embedded chondrocytes as well as growth factors—BMP-2 for its cell-differentiation activity and bFGF for its cell-proliferation activity.

## Materials and methods

### Isolation of chondrocytes

Articular cartilage slices were taken from hip, knee, and shoulder joints of seven 5-week-old Wistar rats for each single experimental set-up. We used 28 rats in all 4 experimental set-ups. Chondrocytes were isolated using 0.1% trypsin with ethylene diamine tetraacetic acid (EDTA) in phosphate buffered saline (PBS) for 20 min and then 0.25% collagenase (Worthington Biochemical, Lakewood, NJ) in Ham's F12 medium (MP Biomedicals, Solon, OH) supplemented with 10% fetal bovine serum (FBS) (MP Biomedicals), antibiotics (100 U/mL penicillin, 100 µg/mL streptomycin, and 0.25 µg/mL amphotericin B (MP Biomedicals) for 3 h at 37°C in a culture tube. Chondrocyte suspensions were strained through a 100-µm Cell Strainer (BD Falcon, Bedford, MA). The chondrocytes collected were centrifuged, washed, and resuspended in Ham's F12 medium. The suspended cells were designated non-culture chondrocytes (NCs). The NCs were further prepared for the 4 experimental conditions (Table 1). The number of harvested NCs was approximately 5.0 × 10^5^ cells per rat.

**Table 1.** The 4 experimental conditions used

Experimental condition	Cell source	Culture method	Mechanical stress	Growth factor	Duration of culture
1	NCs	Monolayer	−	−	P0 to P4
2	NCs	3D	−	−	1–5 weeks
3	P0 chondrocytes	3D	+	−	1 week
4	P0 chondrocytes	3D	+	+	1 week

P0: passage 0; NCs: non-culture chondrocytes.

### Experimental condition 1

NCs serving as positive controls were propagated in monolayer culture in a 100-mm diameter culture dish in a humidified atmosphere of 5% CO_2_ at 37°C. The medium was changed every 3 days. On reaching confluence, the cells were passaged by trypsinization using standard procedures. After the initial harvest, the cells were defined as passage 0 (P0) and they were passaged repeatedly from P1 to P4 (n = 7) to characterize the chondrocytic phenotype of NCs and P0 chondrocytes. “n = 7” means the total number of animals used. Immediately after reaching confluence at each passage, the cells were collected for determination of mRNA expression. Throughout monolayer culture from P0 to P4, the cells were seeded at a density of 1.0 × 10^5^ cells/dish. The number of cells harvested was approximately 1.0 × 10^6^ cells/dish.

### Experimental condition 2

**Figure 1.**
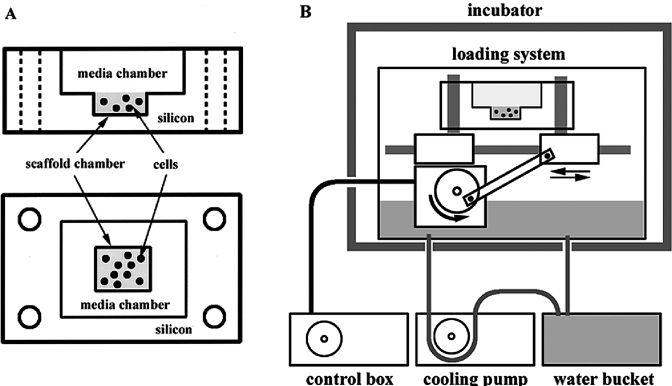
A. The chamber was fixed to the brackets by 4 hook-pins. The cell-seeded collagen gel was placed in the scaffold chamber. The media chamber was filled with serum-free medium with or without growth factor. B. Mechanical loading system. Dynamic compression loading was applied to the cell-seeded scaffold in the chamber.

8 volumes of 3% type I collagen gel (Atelocollagen gel; Koken, Tokyo, Japan) were added to 1 volume of 10-times concentrated serum-free Ham's F12 medium and 1 volume of reconstitution buffer (260 mM NaHCO_3_, 50 mM NaOH, and 200 mM HEPES) with gentle mixing at 0°C. NCs were embedded in the gel mixture and the final cell density was adjusted to 1.0 × 10^6^ cells/mL. The cell-seeded scaffold, the total volume of which was approximately 0.2 mL, was placed in the scaffold chamber (Figure 1A). The cells embedded in the 3D scaffold were cultured in serum-free medium with L-ascorbic acid (50 µg/mL) in an atmosphere of 5% CO_2_ at 37°C for 1–5 weeks (n = 5). The medium was changed every 3 days. In order to characterize the effect of 3D-embedding on preservation of the chondrocytic phenotype, the scaffolds were subjected to real-time RT-PCR and histological analysis at weekly intervals.

### Experimental condition 3

P0 chondrocytes were 3D-embedded with the final cell density adjusted to 1.0 × 10^6^ cells/mL. The 3D-embedded chondrocytes were cultured with serum-free medium under mechanical stress for 1 week. Mechanical stress was applied using the Cell Stretcher System NS 500 (Scholar Tech, Osaka, Japan) (Figure 1B) for 4 durations: 0 (serving as negative control), 10, 60, and 120 min/day (n = 5). The mechanical loading was a cyclic compression, and the amplitude and frequency of compression followed previous studies and was adjusted for 5% strain (Eckstein et al. 1999), 0.33Hz (Korver et al. 1992, Elder et al. 2001). The 3D gel was loaded with a cyclic stress of 2.35 kilopascals (kPa) at its peak stress (1 kPa = 1.0 × 10^3^ N/m^2^). These cells were subjected to 1 loading session per day for 1 week. The first loading session was started 24 h after 3D-embedding of the cells into the collagen. The culture medium was changed every 3 days. Immediately after the final mechanical loading session, each scaffold was removed for extraction of total RNA and histological analysis. In order to determine the optimal mechanical loading for reactivation of the chondrocytic phenotype, the scaffolds were subjected to real-time RT-PCR and histological analysis.

### Experimental condition 4

P0 chondrocytes were 3D-embedded with final cell density adjusted to 1.0 × 10^6^ cells/mL. The 3D-embedded chondrocytes were cultured both under mechanical stress and with growth factor for 1 week. Mechanical stress was applied for 60 min/day at the same degree of strain (5%, 0.33Hz, 2.35kPa) as for experimental condition 3. One of 2 growth factors was included: bFGF (100 ng/mL) (Mandl et al. 2004) or BMP-2 (Sailor et al. 1996, Gründer et al. 2004) (100 ng/mL) (n = 7 for each experiment). The growth factor was added to the serum-free medium in the media chamber (Figure 1A). An experimental group (growth factor (–), mechanical stress (–)) served as negative control. In addition, 2 types of control groups (growth factor (–), mechanical stress (+) serving as “factor control” and growth factor (+), mechanical stress (–) serving as “stress control”) were also prepared to compare with the study group (growth factor (+), mechanical stress (+)). The culture medium was changed every 3 days. Immediately after the final session of mechanical loading, the scaffold was removed for extraction of total RNA and histological analysis. In order to determine the synergistic effect of growth factor and mechanical loading on reactivation of the chondrocytic phenotype, the scaffolds were subjected to real-time RT-PCR and histological analysis. Basic FGF and BMP-2 were purchased from Sigma (Saint Louis, MO).

### RNA extraction and reverse transcription

Total RNA was extracted with the NucleoSpin RNA L kit (Macherey-Nagel, Germany) according to the manufacturer's protocol, and eluted in PCR-grade water. The collected samples were stored at −80°C. RNA was reverse transcribed into single-stranded cDNA with the Transcriptor First Strand cDNA Synthesis kit (Roche Diagnostics) according to the manufacturer's protocol. RNA, anchored-oligo(dT)18 primer, and PCR-grade water were incubated at 65°C for 10 min. Moreover, Transcriptor reverse transcriptase (RT) reaction buffer (Roche), Protector RNase inhibitor (Roche), deoxynucleotide mixture (Roche), and Transcriptor reverse transcriptase (Roche) were added to the samples and incubated at 55°C for 30 min. The reaction was stopped by incubating the samples at 85°C for 5 min. The cDNA pellet was eluted in PCR-grade water and stored at −30°C.

### Quantitative real-time PCR

Real-time PCR was performed with the LightCycler System (Roche Diagnostics) using LightCycler FastStart DNA Master Hybridization Probes (Roche Diagnostics) following the manufacturer's protocol. The reaction was performed in a 20-µL mixture containing 5 µL of the above cDNA and 15 µL master mix. For aggrecan (AGC), type II collagen (Col2), type I collagen (Col1), and glyceraldehyde-3-phosphate dehydrogenase (GAPDH), each cDNA sample was amplified using specific primers and probes (Table 2, Nihon Gene Laboratories Inc., Japan). After an initial denaturation step at 95°C for 10 min, amplification was performed using 50 cycles of denaturation (95°C for 10 s), annealing (60–62°C for 15 s) and extension (72°C for 8–10 s). For each run, a standard curve was generated from purified cDNA of the gene concerned, ranging from 4 × 10^4^ copies to 4 copies. To normalize mRNA levels, we amplified the GAPDH housekeeping gene as an internal control. Gene expression for AGC, Col2, and Col1 was normalized against that for GAPDH.

**Table 2.** Primer sequences and product sizes in real-time PCR

Genes		Sequence of primers (5'-3')	AS (bp)	AT (degrees)
Type II collagen	F	CCCAGAACATCACCTACCAC	201	62
	R	GGTACTCGATGATGGTCTTG		
Aggrecan	F	GATGTCCCCTGCAATTACCA	230	60
	R	TCTGTGCAAGTGATTCGAGG		
Type I collagen	F	TGCCGTGACCTCAAGATGT	181	62
	R	TGGGGTTTGGGCTGATGTA		
GAPDH	F	TGAACGGGAAGCTCACTGG	307	60
	R	TCCACCACCCTGTTGCTGTA		

F: forward; R: reverse; AS: amplicon size; bp: base pairs; AT: annealing temperature.

### Histological analysis

The cell-seeded collagen gel from each group at each culture time point was fixed in 4% formaldehyde in PBS at room temperature for 2 days. These fixed specimens were embedded in paraffin. Each specimen was cut with a microtome into 6-µm thick sections and deparaffinized, rehydrated, and stained with toluidine blue (TB) (0.05% in distilled H_2_O, pH 7.0) to visualize cartilage matrix components.

### Statistics

Results are expressed as mean with 95% confidence limits. Differences between groups were examined with one-factor analysis of variance (ANOVA) using Bonferroni/Dunn post hoc tests. Differences between means were considered statistically significant for p-values < 0.05.

## Results

### Experimental condition 1

**Figure 2.**
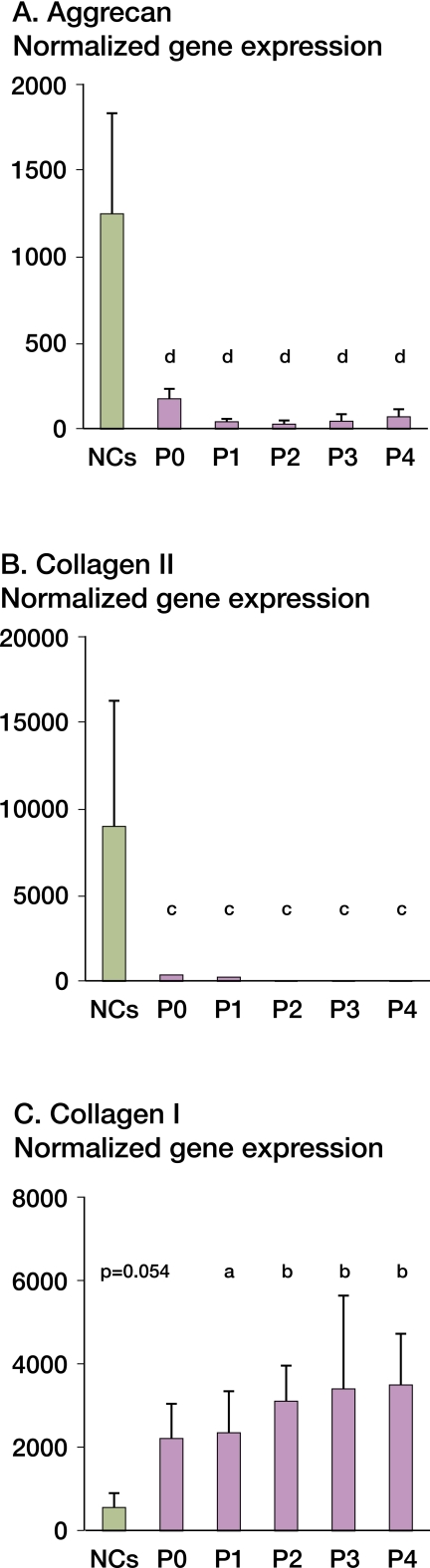
Effects of monolayer culture and repeated passage on aggrecan, collagen II, and collagen I gene expression of chondrocytes (relative to GAPDH gene expression). The monolayer-expanded chondrocytes were rapidly and significantly dedifferentiated from NCs to P0 cells for both aggrecan (panel A) and collagen II (panel B). The chondrocytic phenotypes were attenuated immediately in the P0 cells, with mRNA expression becoming progressively reduced with passage. Results are expressed as mean (95% confidence limit); n = 7. Comparison of mean values was performed by one-factor ANOVA analysis.P0: passage 0; NCs: non-culture chondrocytes. ^a^ p < 0.05, ^b^ p < 0.01, ^c^ p < 0.001, ^d^ p < 0.0001 vs. control (NCs).

In the present study we first characterized the non-culture chondrocytes (NCs) and passage 0 (P0) chondrocytes and compared them with cells that underwent multiple passages (P1 to P4). In terms of aggrecan (AGC) and type II collagen (Col2) mRNA expression, the chondrocytic phenotypes were even attenuated in P0 cells and expression became progressively reduced with passage. The initial downregulation of mRNA expression seen to occur from NCs to P0 cells was statistically significant for both AGC (Figure 2A) and Col2 (Figure 2B).

In contrast, expression of type I collagen (Col1) mRNA was rapidly upregulated from NCs to P0 cells, and it increased progressively with passage. The upregulation of Col1 expression became statistically significant at P1 (p < 0.05). Although there was no statistically significant difference in Col1 expression between NCs and P0 cells (p = 0.054), the upregulation seen was rapid (Figure 2C).

### Experimental condition 2

**Figure 3.**
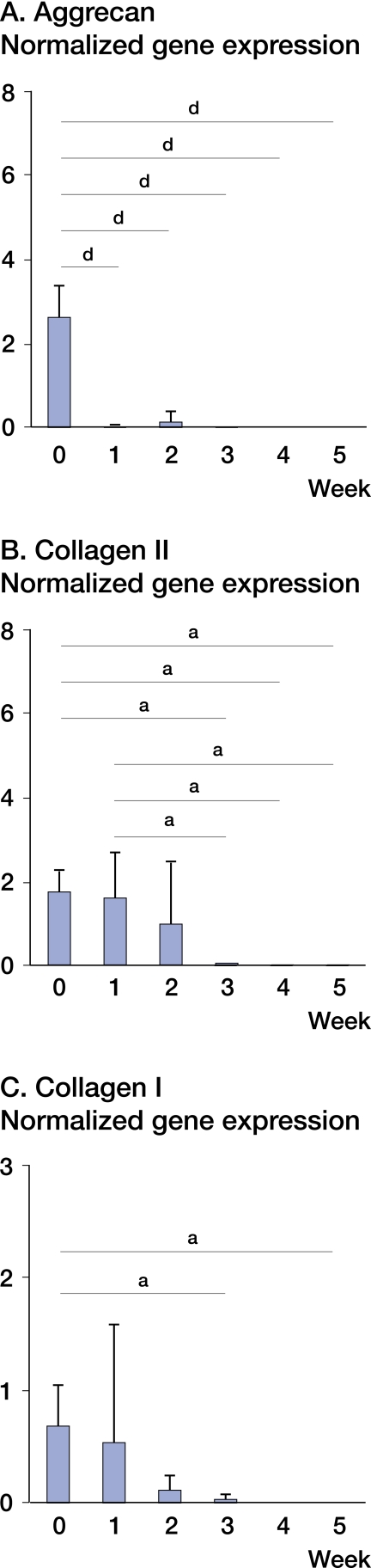
Effects of 3-dimensional (3D) culture for 0–5 weeks on aggrecan, collagen II, and collagen I gene expression of NCs (relative to GAPDH gene expression). Expression of all 3 genes was downregulated during weeks 2–5. Results are expressed as mean (95% confidence limit); n = 5. Comparison of mean values was performed by one-factor ANOVA analysis. NCs: non-culture chondrocytes. ^a^ p < 0.05, ^d^ p < 0.0001.

A variety of attempts to retain the chondrocytic phenotype have been made in the past, and chondrocytes embedded in a 3D collagen scaffold have been suggested to maintain the chondrocytic phenotype for a relatively long time (Kimura et al. 1984, Uchio et al. 2000, Chaipinyo et al. 2004). We then characterized the chondrocytes that were embedded in the 3D collagen scaffold immediately after cell isolation. Unlike the chondrocytes prepared as monolayers, the 3D-embedded chondrocytes maintained their Col2 expression until the third week (Figure 3B).

**Figure 4.**
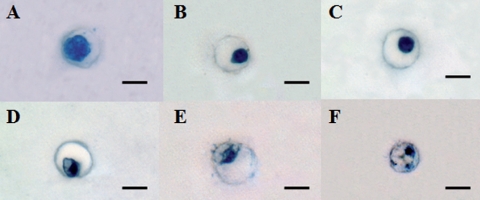
Effects of 3D collagen gel culture. Histological appearance of cells at: week 0 (A), week 1 (B), week 2 (C), week 3 (D), week 4 (E), and week 5 (F). Representative sections were stained with toluidine blue. Scale bars: 10 µm.

AGC mRNA expression was, however, statistically significantly attenuated even after the first week (Figure 3A). Col2 mRNA expression was also attenuated after the third week (Figure 3B). Intriguingly, not only the AGC and Col2 genes but also the Col1 gene became downregulated over the subsequent weeks (fourth and fifth weeks). We also found that GAPDH mRNA expression from the housekeeping gene used as an internal control was also reduced (data not shown). Thus, the total number of viable cells apparently decreased during the fourth and fifth weeks. Histological analysis demonstrated that the cell body in the 3D gel became smaller with time (Figure 4). On the other hand, the ECM (stained with toluidine blue) was not altered in any of the experimental groups (Figure 4).

### Experimental condition 3

**Figure 5.**
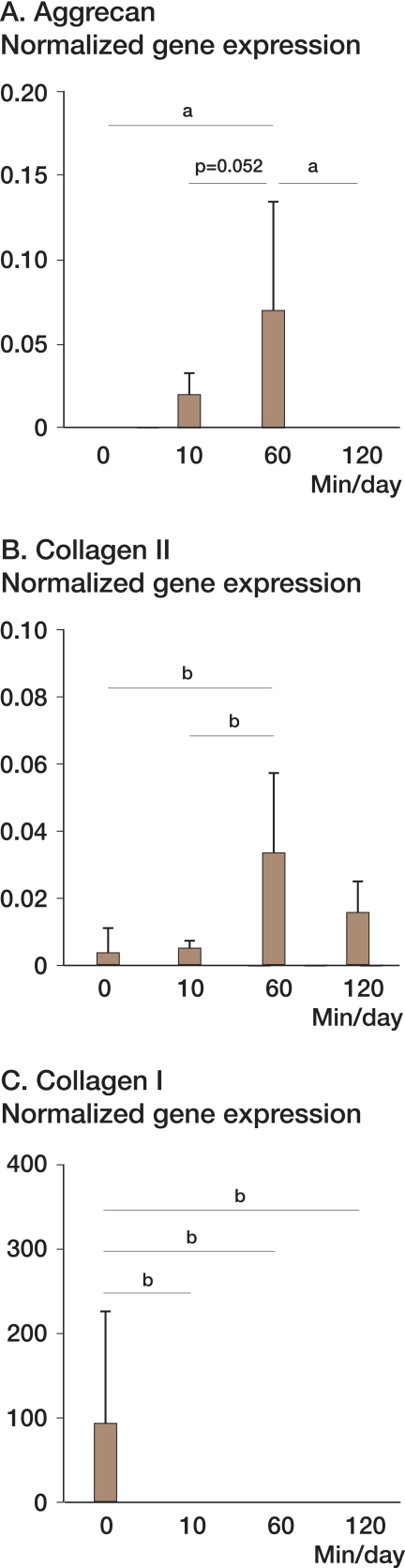
Effects of dynamic compressive loading in serum-free medium on aggrecan, collagen II, and collagen I gene expression of chondrocytes in 3D scaffold (relative to GAPDH gene expression). Of the 4 different durations of loading, 60 min/day gave the greatest effect on aggrecan and collagen II gene expression. Results are expressed as mean (95% confidence limit); n = 5. Comparison of mean values was performed by one-factor ANOVA analysis. ^a^ p < 0.05, ^b^ p < 0.01.

We had thus confirmed that propagation of the chondrocytes in monolayer culture does not allow them to maintain chondrocytic phenotype (Figure 2). In addition, 3D-embedded chondrocytes could not maintain the phenotype of freshly isolated chondrocytes for more than 3 weeks (Figure 3). The present series of experiments was an attempt to obtain a large number of cultured chondrocytes with their chondrocytic phenotype maintained by applying mechanical loading to cells embedded in a 3D scaffold. Dedifferentiated chondrocytes propagated in monolayer culture without any mechanical loading served as negative controls (i.e. loading of 0 min/day; Figure 5). In accordance with our hypothesis, mechanical loading clearly stimulated dedifferentiated chondrocytes. The expression of AGC mRNA was significantly upregulated with loading of 60 min/day as compared to that with loading of 0 and 120 min/day (p < 0.05) (Figure 5A). Col2 mRNA expression was also upregulated with loading of 60 min/day as compared to that with loading of 0 and 10 min/day (p < 0.01) (Figure 5B). On the other hand, Col1 mRNA expression was downregulated with loading of 10, 60, and 120 min/day as compared to that with loading of 0 min/day (p < 0.01) (Figure 5C).

**Figure 6.**
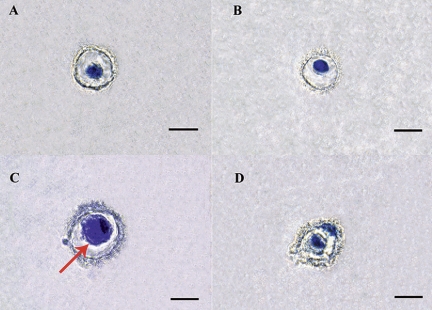
Effects of dynamic compressive loading. Histological appearance of cells treated loading for 0 min/day (A), 10 min/day (B), 60min/day (C) and 120 min/day (D). Toluidine blue staining. The arrow in panel C points to a larger oval cell body. Scale bars: 10 µm.

Histological analysis demonstrated that cells that were mechanically stressed for 60 min/day were characterized by a larger oval cell body than cells that were stressed for 0, 10, or 120 min/day (Figure 6, arrow). In particular, the cells that were stressed for 120 min/day were often serrated, suggesting inactivation of protein production and perhaps cell degeneration. Of all the experimental groups, the ECM area stained with toluidine blue (TB) was broadest in cells with the mechanical loading for 60 min/day, suggesting the most active proteoglycan production (Figure 6).

### Experimental condition 4

Experimental condition 3 showed that mechanical loading of 60 min/day resulted in the most beneficial chondrocytic phenotype (Figures 5 and 6). We thus hypothesized that growth factor would augment expression of the chondrocytic phenotype when added at the same time as mechanical loading.

**Figure 7.**
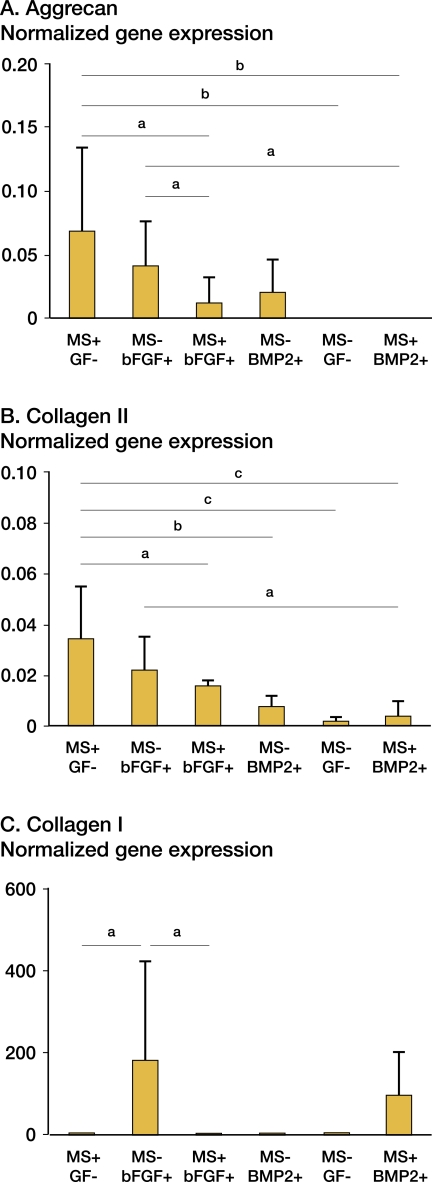
Effects of dynamic compressive loading (for 60 min/day) in combination with growth factor (100 ng/mL bFGF or 100 ng/mL BMP-2) on aggrecan, collagen II, and collagen I gene expression of chondrocytes in 3D scaffold (relative to GAPDH gene expression). These growth factors did not have a synergistic effect under dynamic compressive loading. Results are expressed as mean (95% confidence limit); n = 7. Comparison of mean values was performed by one-factor ANOVA analysis. MS: mechanical stress; GF: growth factor. ^a^ p < 0.05, ^b^ p < 0.01, ^c^ p < 0.001.

First of all, it was clear that application of bFGF or BMP-2 enhanced expression of both AGC mRNA and Col2 mRNA in comparison to the negative control (no mechanical stress and no growth factor). The enhancement of AGC and Col2 gene expression was more prominent in the bFGF-treated cells than in the BMP-2-treated cells (Figures 7A and B). Interestingly, bFGF also enhanced Col1 gene expression (Figure 7C).

In a contrast to our initial expectations, the simultaneous application of growth factor and mechanical stress (study group) suppressed AGC and Col2 gene expression relative to expression of these genes in the “stress control” (growth factor but no mechanical stress). These observations may indicate that chondrocytic differentiation promoted by the mechanical stress may not coexist with cell proliferation driven by the growth factors. The suppression of AGC and Col2 gene expression was more prominent in the bFGF-treated cells than in the BMP-2-treated cells (Figures 7A and B).

In contrast to our initial expectations, simultaneous application of growth factor and mechanical stress also suppressed AGC and Col2 gene expression as compared to the “factor control” (with mechanical stress but no growth factor). These observations may indicate that the cell proliferation driven by growth factors cancels the differentiation promoted by mechanical stress.

**Figure 8.**
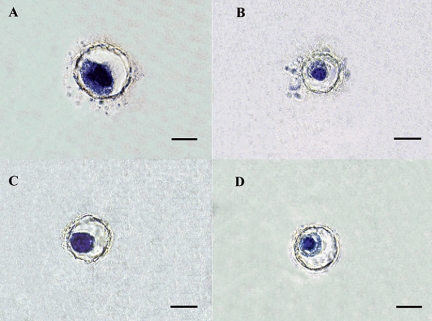
Effects of dynamic compressive loading in combination with growth factor. Histological appearance of cells treated with: A. bFGF but no mechanical stress, B. BMP-2 but no mechanical stress, (C) bFGF and mechanical stress, and (D) BMP-2 and mechanical stress. Toluidine blue staining. The oval cell body and the ECM area in the “stress control” (growth factor but no mechanical stress, corresponding to panels A and B) were generally larger than in each study group (corresponding to panels C and D). Scale bars: 10 µm.

In accordance with the gene expression results, the histological analysis demonstrated that the cells in the “stress controls” (with growth factor but no mechanical stress) (Figure 8A and B) were characterized by a larger oval cell body than that of the cells in the study group, which received both growth factor and mechanical stress (Figure 8C and D). The ECM stained with TB was broader in the “stress control” (growth factor but no mechanical stress) (Figure 8A and B) than in the negative control (no growth factor and no mechanical stress) (Figure 6A) and each study group (growth factor and mechanical stress) (Figure 8C and D). Of all these experimental groups, the ECM was largest in the “factor control” (with mechanical stress but no growth factor) (Figure 6A and C, Figure 8).

## Discussion

Autologous chondrocyte implantation (ACI) has received much attention due to its potential as a novel treatment of damaged cartilage (Brittberg et al. 1994). A variety of ACI protocols have been explored, but many of them have used chondrocytes propagated as monolayers. For successful tissue engineering of the transplantation-ready cartilage, a large quantity of chondrocytes must be prepared and propagation of cells in monolayer culture may be required. Numerous investigators have studied the phenotypic changes in chondrocytes during passage in monolayer culture. It has often been reported that the synthesis of the cartilage-specific matrix decreases while the synthesis of the non-cartilage specific matrix proteins, e.g. type I collagen, increases. These phenotypic changes in chondrocytes during monolayer culture, including loss of synthesis of extracellular matrix (ECM), are likely to reflect dedifferentiation (Holtzer et al. 1960, Abbot and Holtzer 1966, Mayne et al. 1976, von der Mark et al. 1977, Benya et al. 1978). In spite of a variety of attempts by other workers, dedifferentiated chondrocytes prepared in monolayer have not been made to re-differentiate back into ECM-synthesizing chondrocytes (Darling et al. 2005). Thus, an alternative scheme to maintain the chondrocytic phenotype would be of great advantage.

In this study, we first characterized passage 0 (P0) chondrocytes and non-culture chondrocytes (NCs), since the information gained about the phenotype of these cells would be used during the rest of our study. Then the chondrocytic phenotypes of NCs and P0 cells were compared with those of the cells that underwent subsequent passages (P1 to P4) (Figure 2). Our study shows that in monolayer culture, dedifferentiation of articular chondrocytes is apparent as early as P0, and that gene expression becomes even more attenuated as the number of passages increases (Figure 2). These data thus reconfirm that chondrocytes propagated in monolayer culture do not retain their phenotype. In turn, it must be remembered that the P0 cells, which were clearly dedifferentiated at this stage (Figure 2), became reactivated by mechanical loading (Figure 5).

Three-dimensional (3D) culture systems have been tested in order to maintain or reactivate the chondrocytic phenotype (Benya and Shaffer 1982, Kimura et al. 1984, Guo et al. 1989, Homminga et al. 1993, Uchio et al. 2000). Some investigators have indeed asserted that 3D culture can allow redifferentiation of dedifferentiated chondrocytes (Benya et al. 1982, Liu et al. 1998, Tallheden et al. 2004). The assertion that implantation of the 3D-embedded chondrocytes can result in better cartilage regeneration also led to a clinical trial (Ochi et al. 2001). The basic research related to this clinical trial showed that freshly isolated chondrocytes embedded in type I collagen gel could proliferate, synthesize ECM, and maintain their chondrocytic phenotype for up to 4 weeks (Chaipinyo et al. 2004). In turn, others using different types of collagen scaffold reported negative effects of 3D-embedded chondrocytes (van Susante et al. 1995). The conflicting data may have resulted from the different collagens used for 3D culture.

In the present study, we used the same collagen (prepared by the same manufacture) as the one used in the study by Chaipinyo et al. (2004). We also confirmed that 3D-embedded chondrocytes retained their expression of type 2 collagen mRNA until the third week. However, it must also be remembered that 3D-embedded chondrocytes retained their chondrocytic phenotype only partially, and only for a limited time (Figure 3). In order to regenerate the transplantation-ready cartilage, the cells must be prepared in large numbers with their chondrocytic phenotype retained. Our observation that the 3D-embedded chondrocytes only partially retain their chondrocytic phenotype, and only for a limited time, caused us to seek for another way of reactivating 3D-embedded chondrocytes.

Mechanical loading has been suggested as an important way of regulating the chondrocytic activities (Broom et al. 1980, Palmoski and Brandt 1984, Schneiderman et al. 1986, Gray et al. 1988, Sah et al. 1989, Korver et al. 1992, Parkkinen et al. 1992, Guilak et al. 1994, Buschmann, et al. 1995, Lee and Bader 1997, Ragan et al. 1999, Elder et al. 2001). In the third series of our experiments, we showed that dynamic compressive loading clearly reactivates dedifferentiated chondrocytes (Figure 5A and B). Application of dynamic compressive load also caused suppression of type I collagen expression, irrespective of the duration of the loading (Figure 5C). Of the different durations of dynamic compressive loading tested, loading of 60 min/day had the best effect on expression of Col2 and AGC mRNA (Figure 5A and B). The nature of our study only allows us to guess the reason for the inferior results with longer duration of treatment, i.e. 120 min/day. The total response of genetic expression may reflect the cumulative magnitude of strain caused by each loading. Mechanical loading of long duration may result in strain on the cell.

In a variety of studies, growth factors have been shown to promote proliferation of chondrocytes and ECM synthesis in vitro and in vivo (Guerne et al. 1994, Sailor et al. 1996, Martin et al. 1999). In the final series of our experiments, we hypothesized that administration of growth factor, i.e. bFGF or BMP-2, at the same time as mechanical loading would further augment the chondrocytic phenotype of the 3D-embedded chondrocytes. However, in clear contrast to our expectations, under mechanical loading these growth factors did not play any synergistic role (Figures 7 and 8).

In contrast to the well-documented positive effects of IGF-1 (Gooch et al. 2001, Bonassar et al. 2001, Mauck et al. 2003) and TGF−β (Mauck et al. 2003) on 3D-embedded chondrocytes, only a few studies have investigated the effects of bFGF (Martin et al. 1999, Shida et al. 1996) or BMP-2 (Martin et al. 2001) on 3D-embedded chondrocytes, especially under mechanical loading. However, from the present data we should probably not attribute the negative effects of these factors applied simultaneously with mechanical loading simply to the different conditions of the chondrocyte culture, as attenuated phenotypic expression was confirmed not only against the “factor control” but also against the “stress control” (Figures 7 and 8).

The simultaneous application of two treatments suppressed the chondrocytic phenotype relative to the “factor control” (mechanical stress but no growth factor), suggesting that the cell proliferation driven by the growth factor cancelled the differentiation promoted by the mechanical stress. In turn, the simultaneous application also suppressed chondrocytic phenotype relative to the “stress control” (growth factor but no mechanical stress), suggesting that the chondrocytic differentiation promoted by the mechanical stress may not coexist with cell proliferation driven by the growth factors. Application of these growth factors at different time points during regeneration of the cartilage may result in better chondrocytic phenotype.

In summary, the chondrocytes cultured as monolayers rapidly dedifferentiated, and even the 3D-embedded chondrocytes could not maintain the chondrocytic phenotype for a long time. Mechanical loading clearly reactivated expression of the aggrecan and type II collagen genes at loading of 60 min/day compared to the other durations of loading. BMP-2 and bFGF also increased aggrecan and type II collagen mRNA expression when applied independently. Unlike previous reports using monolayer cultures of chondrocytes, however, BMP-2 or bFGF did not augment the chondrocytic phenotype when applied simultaneously with mechanical loading. This result suggests that application of mechanical loading and growth factors administered at different time points may contribute to regeneration of the transplantation-ready cartilage, resulting in better chondrocytic phenotype.
